# The Farthest Color Voronoi Diagram in the Plane

**DOI:** 10.1007/s00453-025-01311-1

**Published:** 2025-06-06

**Authors:** Ioannis Mantas, Evanthia Papadopoulou, Rodrigo I. Silveira, Zeyu Wang

**Affiliations:** 1 Faculty of Informatics, Università della Svizzera italiana, Lugano, Switzerland; 2 Departament de Matemàtiques, Universitat Politècnica de Catalunya, Barcelona, Spain

**Keywords:** Farthest-site Voronoi diagram, Color Voronoi diagram, Point clusters, Color spanning disk, Straddles, Divide and conquer

## Abstract

The farthest-color Voronoi diagram (FCVD) is defined on a set of *n* points in the plane, where each point is labeled with one of *m* colors. The colored points constitute a family $\mathcal {P}$ of *m* clusters (sets) of points in the plane whose farthest-site Voronoi diagram is the FCVD. The diagram finds applications in problems related to facility location, shape matching, data imprecision, and others. In this paper we present structural properties of the FCVD, refine its combinatorial complexity bounds, and present efficient algorithms for its construction. We show that the complexity of the diagram is $O(n\alpha (m)+\textit{str}(\mathcal {P}))$, where $\textit{str}(\mathcal {P})$ is a parameter reflecting the number of *straddles* between pairs of clusters, which is $O(m(n-m))$. The bound reduces to $O(n+ \textit{str}(\mathcal {P}))$ if the clusters are pairwise *non-crossing*. We also present a lower bound, establishing that the complexity of the FCVD can be $\Omega (n+m^2)$, even if the clusters have pairwise disjoint convex hulls. Our algorithm runs in $O((n+\textit{str}(\mathcal {P}))\log ^3 n)$-time, and in certain special cases in $O(n\log n)$ time.

## Introduction

Voronoi diagrams are among the most influential structures in computational geometry. Given a set *S* of *n* simple geometric objects in a space, called sites, the classic *nearest-neighbor* Voronoi diagram of *S* subdivides the underlying space into maximal regions such that all points within one region share the same nearest site. In the classic *farthest-site* variant, all points within one region have the same farthest site. Numerous generalizations of this simple concept have been investigated, motivated by applications across a wide range of scientific fields. For an extensive coverage of Voronoi diagrams, their structural properties and generalizations, see the book of Aurenhammer et al. [6]; see also the book of Okabe et al. [25] for a wealth of applications.

In this paper, we consider *color Voronoi diagrams* for a set *S* of *n* point sites in the plane, which have been assigned an additional label, a color, representing a common property that points of the same color share. A maximal set of points with the same color assignment is called a *cluster*. The color assignment transforms *S* into a family $\mathcal {P}$ of *m* clusters (sets) of points in the plane, where each cluster corresponds to one of the *m* colors. The distance between a point $x\in \mathbb {R}^2$ and a cluster $P$ is defined by the minimum among the distances from *x* to any point in $P$.

**Fig. 1:**
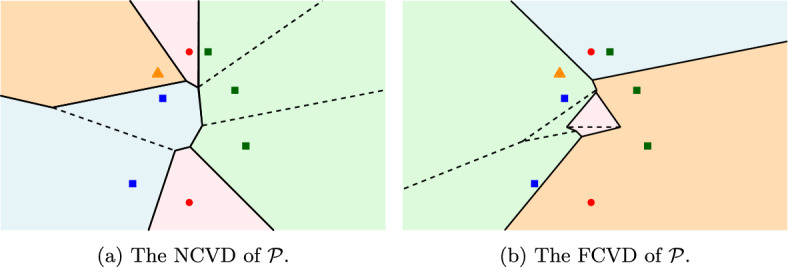
Color Voronoi diagrams of a family $\mathcal {P}$ of $m=4$ clusters of $n=8$ points. Regions are filled with the color of their corresponding cluster. The dashed edges indicate the internal skeletons

The *nearest color Voronoi diagram* (NCVD) and the *farthest color Voronoi diagram* (FCVD) of the family $\mathcal {P}$ can be defined in a natural way: the *nearest* (resp., *farthest*) *color Voronoi region* of a cluster $P$ is the locus of points closer to (resp., farther from) $P$ than to any other cluster in $\mathcal {P}$. These regions and their respective boundaries reveal the two diagrams, the NCVD and the FCVD of $\mathcal {P}$, see Fig. 1.

Although naturally defined, the two diagrams have a different relation to their classic (uncolored) counterparts. The NCVD is a “min–min” type of diagram, which can be directly obtained from the ordinary nearest-neighbor Voronoi diagram of *S*, and whose complexity is naturally *O*(*n*). In contrast, the FCVD is a “max–min” type of diagram that bears no relation to the farthest-site Voronoi diagram of *S*. It was first studied by Huttenlocher et al. [18] as the upper envelope of *m* *Voronoi surfaces* in $\mathbb {R}^3$, showing that its combinatorial complexity is $O(mn\alpha (mn))$, and $\Omega (mn)$ in the worst case, where $\alpha ()$ is the extremely slowly-growing inverse of Ackermann’s function. The upper bound was later settled to *O*(*mn*) by Abellanas et al. [1].

Another variant of color Voronoi diagrams is the so called *Hausdorff Voronoi diagram* (HVD) of the family $\mathcal {P}$, which is a “min–max” type of diagram, also known as the cluster Voronoi diagram of $\mathcal {P}$. In the HVD, the distance between a point $x\in \mathbb {R}^2$ and a cluster $P$ is the maximum distance between *x* and any point in $P$, and Voronoi regions are defined in the nearest sense. The HVD has been extensively studied, see e.g., [4, 14, 19, 26, 29]. In this paper we focus on the FCVD.

Color Voronoi diagrams have been motivated by diverse applications. Consider the following typical problem in facility location: given the locations of multiple types of facilities (e.g., hospitals, schools, etc.), where each location is a point and each type of facility is a color, find the point (a potential residence location) whose distance to all the facilities is minimized. This point is the center of the *minimum color spanning disk* [1], a disk that contains a point of each facility type, which is a point on the FCVD of the facility locations. In fact, the FCVD provides more information: it creates a planar map which encodes the minimum color spanning disk for every point in the plane. This map can be particularly useful to facility location problems. The problem of computing minimum color spanning disks arises also in spatial databases, see e.g., [11, 17].

The FCVD is useful in shape matching, as it can be used to efficiently compute the translation that minimizes the Hausdorff distance between two point sets [18]. It is also useful in handling imprecision in geometric data as point clusters can represent the set of possible locations of an object whose exact location is unknown [20]. In [2], the FCVD is used as a tool to solve the minimum color spanning disk problem of imprecise points. Furthermore, it has been used to solve variants of the Steiner tree problem [8], and in sensor deployment in wireless sensor networks [22]. Finally, point clusters appear in classification problems where a query point is assigned a color as inferred by its neighbors. In two dimensions, queries involved in the *nearest-neighbor classification rule* can be efficiently answered by the NCVD, which can be constructed in an output-sensitive way as shown by Bremner et al. [10].

The HVD, on the other hand, has been motivated by geometric scenarios related to predicting and evaluating faults in geometric networks. In particular, the HVD has found direct applications in evaluating the *critical area* of VLSI designs, see e.g., [27] and references therein, or [31] for an industrial CAD tool in VLSI manufacturing. Both diagrams have been combined to compute special cases of stabbing circles for parallel line segments [12].

The FCVD can be computed in $O(mn \log n)$ time by computing the upper envelope of *m* *Voronoi surfaces* in $\mathbb {R}^3$ [18]. It can also be seen as the upper envelope of a family of lower envelopes of planes in $\mathbb {R}^3$, where each lower envelope corresponds to a cluster in $\mathcal {P}$, using the standard lifting transformation to the unit paraboloid in $\mathbb {R}^3$ [16]. Then, it can be computed in $O(n^2)$-time by applying the divide and conquer technique of Edelsbrunner et al. [14] to compute the upper envelope of piecewise linear functions. This is optimal in the worst case when the diagram has complexity $\Theta (n^2)$, however, the algorithm remains quadratic, even if the complexity of the diagram is *O*(*n*). Similarly, the running time of the algorithm of Huttenlocher et al. [18] remains $\Theta (mn\log n)$, even if the computed envelope has complexity *O*(*n*). Some restricted instances admitting diagrams of *O*(*n*) complexity have been considered by Bae [7], Claverol et al. [12] and Iacono et al. [19].

The potentially quadratic size and time complexity to compute the FCVD can constitute an obstacle to its use in applications where the input size is large. However, there are many situations in which the diagrams have subquadratic complexity. This leads to two relevant challenges: (1) understanding the conditions that can result in an FCVD of quadratic complexity; (2) computing the FCVD in output-sensitive time, so that quadratic time is incurred only when the output size requires it. In this paper we take steps in these two directions by stating the reasons behind the potential superlinear behavior of the FCVD, and presenting algorithms whose complexity depends on a new parameter that quantifies these reasons.

### Contribution

In this paper we present structural properties of the FCVD, refine its combinatorial complexity bounds, and present efficient algorithms for its construction. We show that the complexity of the diagram is $O(n\alpha (m)+\textit{str}(\mathcal {P}))$, where $\textit{str}(\mathcal {P})$ is a new parameter reflecting the number of *straddles* between pairs of clusters, which is $O(m(n-m))$. If the clusters are pairwise *non-crossing*, i.e., their convex hulls admit at most two supporting segments, then the bound reduces to $O(n+ \textit{str}(\mathcal {P}))$. A cluster $Q$, in particular, a pair of points $q_1,q_2\in Q$, is said to *straddle* a pair of points $p_1,p_2\in P$, if the disks through $(p_1,p_2,q_1)$ and $(p_1,p_2,q_2)$ are empty of points in $P$ and $Q$ (see Definition 4) in which case the line segment $q_1q_2$ intersects (straddles) the line through $p_1,p_2$, see Fig. 6. A straddle is a relaxed version of the notion of a crossing between $q_1q_2$ and $p_1p_2$, and may cause the split of the FCVD region of *Q* in two different faces. Straddles are the reason behind the potential quadratic behavior of the FCVD, which is caused by the occurence of multiple bounded faces associated with single clusters.

In particular, the term $O(n\alpha (m))$ bounds the number of unbounded faces of $FCVD (\mathcal {P})$ and the same bound applies to the unbounded faces of $HVD (\mathcal {P})$. The term $O(n+ \textit{str}(\mathcal {P}))$ bounds the number of bounded faces of $FCVD (\mathcal {P})$. Both bounds are tight in the worst case. Note that $\textit{str}(\mathcal {P})=O(m(n-m))$, which refines but does not contradict the $\Omega (mn)$ lower bound of [18] as the two coincide in the worst case when $m=\Theta (n)$. We also give sufficient conditions under which the FCVD has complexity *O*(*n*) as well as conditions under which the FCVD falls under the framework of abstract Voronoi diagrams.

Furthermore, we establish a lower bound by showing that the FCVD may have complexity $\Omega (n+m^2)$, even if the clusters in $\mathcal {P}$ are *linearly separable*, i.e., their convex hulls are pairwise disjoint. We do this by presenting a family of clusters, each composed of two points, that generate $\Theta (m^2)$ straddles, resulting in a quadratic-size FCVD. The construction, and especially its complexity analysis, is involved, however, it is relevant and even surprising because it shows a sharp contrast to the HVD, for which linear separability guarantees the linear complexity of the diagram.

Finally, we present an $O((n+\textit{str}(\mathcal {P}))\log ^3 n)$-time construction algorithm, which is considerably more efficient than previous approaches, if $\textit{str}(\mathcal {P})$ is small compared to *mn*. For certain special cases where the FCVD has *O*(*n*) complexity, we show how to compute the diagram in optimal $O(n\log n)$ time.

#### Outline.

The paper is organized as follows. In Sect. 2, we give the necessary preliminaries, and in Sect. 3, we study the structural properties of the diagram. In Sect. 4, we study conditions under which the diagram has *O*(*n*) complexity, and in Sect. 5 we provide a lower bound indicating that the complexity of the diagram can be quadratic, even if the clusters are pairwise linearly separable. In Sect. 6 we present efficient algorithms to construct the FCVD. We conclude the paper in Sect. 7.

## Preliminaries

Let $\mathcal {P}$ be a family of *m* clusters (sets) of points in $\mathbb {R}^2$, denoted $P_1,\dots ,P_m$, for $m>1$, where the total number of points is *n*. The set of all points is $S = \bigcup _{1 \le i \le m} P_i$. Each point in $P_i$ is assigned *color* *i*. To keep the presentation simple, we make a general position assumption that no three points are collinear and no four points are co-circular.

We use the following notation. For two points *p*, *q* in the plane, denote by *d*(*p*, *q*) the Euclidean distance between *p* and *q*, by *L*(*p*, *q*) the line through *p* and *q*, by $\overline{pq}$ the line segment with endpoints *p* and *q*, and by *b*(*p*, *q*) the Euclidean bisector of *p* and *q* (i.e., the set of points in the plane equally distant from *p* and *q*). Denote by *C*(*p*, *q*, *r*) the circle through points *p*, *q* and *r*, and by *D*(*p*, *q*, *r*) the corresponding disk, which is considered an open set. The convex hull of a set of points $P$ is denoted by $CH (P)$. Given a region $f \subset \mathbb {R}^2$, we denote by $\partial f$ its boundary, and by $\overline{f}$ its closure.

The ordinary Voronoi diagram of a set of points $P$ is a plane graph that partitions the plane into regions such that the Voronoi region of a point $p \in P$ is

$$\begin{aligned}&\textit{reg}(p,P) := \{x \in \mathbb {R}^2 \, | \, d(x,p) < d(x,q), \ \forall q \in P\setminus \{ p \} \}. \end{aligned}$$

The Voronoi diagram of $P$ is

$$\begin{aligned}&\mathcal {V}(P) := \mathbb {R}^2 \setminus \bigcup _{p \in P} \textit{reg}(p,P). \end{aligned}$$

In this paper we often refer to $\mathcal {V}(P)$ as the *skeleton of* $P$.

Two clusters $P$ and $Q$ are called *non-crossing* if their convex hull $CH (P\cup Q)$ contains exactly two edges with one endpoint in $P$ and one in $Q$. If their convex hulls are disjoint, then $P$ and $Q$ are called *linearly separable*. $P$ and $Q$ are called *non-enclosing* if none of them is enclosed in the convex hull of the other. A family of clusters $\mathcal {P}$ is called non-crossing (resp. linearly separable or non-enclosing) if all clusters are pairwise non-crossing (resp. linearly separable or non-enclosing).

### Definition 1

The (minimum) distance of a point $x \in \mathbb {R}^2$ to a cluster $P$ is

$$\begin{aligned}&d_c(x,P) := \min \limits _{p \in P} d(x,p). \end{aligned}$$

The *color bisector* of two clusters $P$ and $Q$ is

$$\begin{aligned}&b_c(P,Q) := \{x \in \mathbb {R}^2\, | \, d_c(x,P) = d_c(x,Q)\}. \end{aligned}$$

**Fig. 2:**
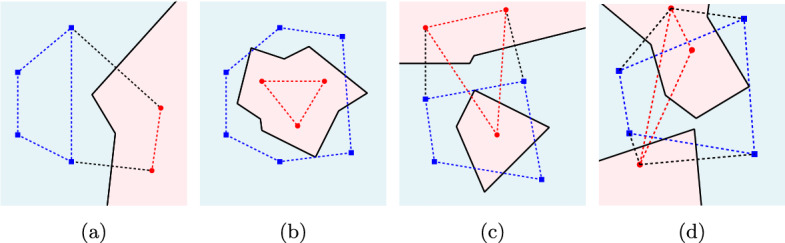
Color bisectors of two clusters $P$ (blue) and $Q$ (red) shown in solid black lines. The dashed edges illustrate the convex hulls. **a** Linearly separable clusters. **b** One cluster enclosed in the convex hull of the other. **c** Non-crossing clusters; note that the color bisector contains a cycle. **d** Crossing clusters

The properties of color bisectors are given in the following proposition, see Fig. 2.

### Proposition 1

The bisector $b_c(P,Q)$ is a subgraph of $\mathcal {V}(P\cup Q)$, which consists of disjoint unbounded chains and cycles. Each unbounded chain corresponds to a distinct pair of convex-hull edges on $CH (P \cup Q)$ that have one endpoint in $P$ and one in $Q$. Furthermore,

1. If $P$ and $Q$ are non-crossing, $b_c(P,Q)$ consists of one unbounded chain and cycles (if any).
2. $b_c(P,Q)$ has no unbounded chains (only cycles) if and only if the convex hull of one cluster is enclosed in the other.

### Proof

By definition, the bisector $b_c(P,Q)$ is a subgraph of $\mathcal {V}(P\cup Q)$. The corresponding Voronoi diagram is a map that covers the plane and can be colored by two colors, where $\textit{reg}(p,P\cup Q)$ is colored blue and $\textit{reg}(q,P\cup Q)$ is colored red, for any $p \in P$ and $q\in Q$. Then the boundary between different color regions is exactly the color bisector $b_c(P,Q)$, thus, it consists of disjoint unbounded chains and cycles. The remaining properties are derived directly from the well-known relation between the unbounded Voronoi edges of $\mathcal {V}(P\cup Q)$ and the convex hull $CH (P\cup Q)$, see e.g., [30]. In particular, it implies that each unbounded component of $b_c(P,Q)$ corresponds to a distinct pair of convex-hull edges in $CH (P \cup Q)$ that have one endpoint in $P$ and one in $Q$. If the clusters are non-crossing, there are exactly two such edges by definition, so there is exactly one unbounded component. This shows claim (1). If there is no unbounded component, then there is no such edge on $CH (P \cup Q)$, which means that one cluster encloses another. This proves claim (2). $\square$

We define the following two *color Voronoi diagrams*.

### Definition 2

The *nearest color Voronoi region of a cluster* $P\in \mathcal {P}$ is

$$\begin{aligned}&{n_{c}reg}(P,\mathcal {P}) := \{x \in \mathbb {R}^2 \, | \, d_c(x,P) < d_c(x,Q), \ \forall Q\in \mathcal {P}\setminus \{ P\} \}. \end{aligned}$$

The *nearest color Voronoi diagram (NCVD)* of $\mathcal {P}$ is

$$\begin{aligned}&NCVD (\mathcal {P}) := \mathbb {R}^2 \setminus \bigcup _{P\in \mathcal {P}} {n_{c}reg}(P,\mathcal {P}). \end{aligned}$$

The NCVD can be easily obtained from the ordinary Voronoi diagram of the set *S* of all points, $\mathcal {V}(S)$: the (nearest) color Voronoi region of a cluster $P$ is the union of all the Voronoi regions of $\mathcal {V}(S)$ that belong to points in $P$, see Fig. 1a. Thus, the $NCVD (\mathcal {P})$ has complexity *O*(*n*) and it can be computed in $O(n\log n)$ time.

### Definition 3

The *farthest color Voronoi region of a cluster* $P\in \mathcal {P}$ is

$$\begin{aligned}&{f_{c}reg}(P,\mathcal {P}) := \{x \in \mathbb {R}^2 \, | \, d_c(x,P) > d_c(x,Q), \ \forall Q\in \mathcal {P}\setminus \{ P\} \}. \end{aligned}$$

The *farthest color region of a point* $p \in P$ is

$$\begin{aligned}&{f_{c}reg}(p,\mathcal {P}) := \{x \in {f_{c}reg}(P,\mathcal {P}) \, | \, d(x,p) < d(x,q), \forall q \in P\setminus \{ p \} \}. \end{aligned}$$

The *internal skeleton of* ${f_{c}reg}(P,\mathcal {P})$ is

$$\begin{aligned}&\mathcal {V}(P) \cap {f_{c}reg}(P,\mathcal {P}). \end{aligned}$$

The *farthest color Voronoi diagram (FCVD)* of $\mathcal {P}$ is

$$\begin{aligned}&FCVD (\mathcal {P}) := \mathbb {R}^2 \setminus \bigcup _{P\in \mathcal {P}} {f_{c}reg}(P,\mathcal {P}); \end{aligned}$$

its augmented version (augmented by the internal skeletons) is

$$\begin{aligned}&FCVD ^{*}(\mathcal {P}) := \mathbb {R}^2 \setminus \bigcup _{p \in S} {f_{c}reg}(p,\mathcal {P}). \end{aligned}$$

**Fig. 3:**
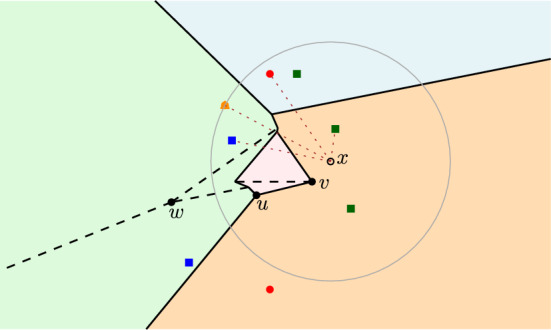
An FCVD of 4 clusters. The dashed edges indicate the internal skeletons of $FCVD ^{*}(\mathcal {P})$. Vertex *u* is a regular Voronoi vertex, *v* is a mixed vertex, and *w* is an internal vertex. The dotted edges illustrate the distance from a point $x \in \mathbb {R}^2$ to each cluster. The gray disk is the farthest color disk of point *x*

The *farthest color disk* of a point $x \in \mathbb {R}^2$ is the disk centered at *x* of radius *d*(*x*, *p*), where $x \in \overline{{f_{c}reg}(p,\mathcal {P})}$. This disk contains in its interior at least one point from every cluster in $\mathcal {P}\setminus \{P\}$ and no point of $P$, for $P$ the cluster containing *p*. The farthest color disk is also known as the *minimum color spanning disk* [1]. See, e.g., the disk centered at point *x* in Fig. 3. The figure also shows an example of $FCVD (\mathcal {P})$ and $FCVD ^{*}(\mathcal {P})$, where the dashed lines depict the internal skeletons.

The FCVD contains various different types of edges and vertices, refer again to Fig. 3. We use the following naming conventions: Voronoi edges that are subsets of color bisectors are called *color Voronoi edges*, while the edges of the internal skeletons of farthest color regions are called *internal edges*. Voronoi vertices that are incident to three color edges are *regular Voronoi vertices*, while vertices incident to two color edges and one internal edge are called *mixed vertices*. Vertices of the internal skeletons are called *internal vertices*.

The FCVD is conceptually related to the Hausdorff Voronoi diagram of $\mathcal {P}$, which is denoted $HVD (\mathcal {P})$. In this diagram, distance between a point and a cluster is measured in the farthest sense, $d_f(x,P):= \max _{p \in P} d(x,p)$, and a Hausdorff Voronoi region is defined in the nearest sense: $\textit{hreg}(P,\mathcal {P}):= \{x \in \mathbb {R}^2 \, | \, d_f(x,P) < d_f(x,Q), \ \forall Q\in \mathcal {P}{\setminus } \{ P\} \}$.

Using the transformation of Edelsbrunner and Seidel [13] in $\mathbb {R}^3$, both diagrams can be viewed as envelopes of *wedges* in 3D: lift up the points of each cluster onto the unit paraboloid, then map each lifted point to its tangent plane; each cluster $P$ is transformed to a set of planes in 3D. The lower (resp., upper) envelope of such a set forms a *lower* (resp., *upper*) *wedge*. The HVD and FCVD correspond to the upper envelope of all lower wedges, and to the lower envelope of all upper wedges, respectively. This transformation was explicitly given for the HVD by Edelsbrunner et al. [14], and it has been pointed out for the FCVD by Claverol et al. [12]. The transformation indicates that the unbounded faces of $HVD (\mathcal {P})$ and $FCVD (\mathcal {P})$ coincide, while they are unbounded in opposite directions.

Next, we review from [29] the definition of a *hull*, which characterizes the unbounded faces of the HVD, and therefore also of the FCVD.

Given the family of clusters $\mathcal {P}$, a point $p \in P$, where $P\in \mathcal {P}$, is a *hull vertex*, if there exists a line $\ell$ through *p* such that $P$ lies entirely in one half-plane defined by $\ell$, and every cluster $Q$ in $\mathcal {P}\setminus \{P\}$ intersects the other half-plane. The segment $\overline{pq}$ connecting two hull vertices $p\in P$ and $q \in Q$ is a *hull edge*, if the line *L*(*p*, *q*) leaves $P$ and $Q$ on one side, and the half-plane of the other side intersects every cluster in $\mathcal {P}\setminus \{ P,Q\}$. Each hull edge $\overline{pq}$ is associated with a unit vector that is normal to $\overline{pq}$; the direction of the unit vector points away from $P,Q$.

**Fig. 4:**
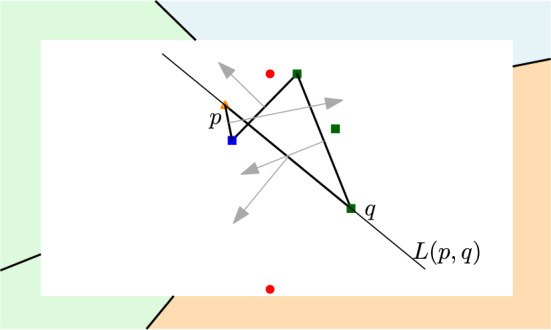
The cluster hull of the example in Fig. 3. The arrows depict the unit vectors normal to hull edges; they correspond to unbounded edges in $FCVD ^{*}(\mathcal {P})$

The sequence of hull edges sorted by the angular ordering of their normal vectors defines a closed (non-simple) polygonal curve, called the *cluster hull*, or simply the *hull*, of $\mathcal {P}$, denoted CLH$(\mathcal {P})$. Refer to Fig. 4.

## Structural properties and complexity

In this section, we point out some structural properties of the farthest color Voronoi diagram. We start with the following *visibility property*.

### Proposition 2

For any point $x \in {f_{c}reg}(p,\mathcal {P})$, let *r* be the ray emanating from *x*, along *L*(*p*, *x*), in the direction away from *p*. Then the region ${f_{c}reg}(p,\mathcal {P})$ must contain the entire segment $r\cap \textit{reg}(p,P)$, where $p\in P$.

This property is analogous to a corresponding visibility property of the HVD [29] and has also been pointed out in [7]. It can be applied to derive the following property.

### Proposition 3

Let *f* be a face of ${f_{c}reg}(P,\mathcal {P})$, and let $T= \mathcal {V}(P)\cap f$ be the internal skeleton of *f*; *T* is incident to the mixed vertices of $\partial f$.

1. If *f* is bounded, then *T* is a tree, which is necessarily non-empty.
2. If *f* is unbounded, then *T* may be empty, in which case $f\subseteq \textit{reg}(p,P)$. If non-empty, it is a forest, where each tree has at least one unbounded edge incident to infinity.

### Proof

Assuming $m>1$, no point $p\in P$ can be contained in *f*; thus, no region $\textit{reg}(p,P)$ can be entirely contained in *f*. Hence, *T* contains no cycle, thus, it must be a tree or a forest. Furthermore, if *T* is empty, then *f* must be entirely contained in $\textit{reg}(p,P)$ for some $p\in P$.

**Fig. 5:**
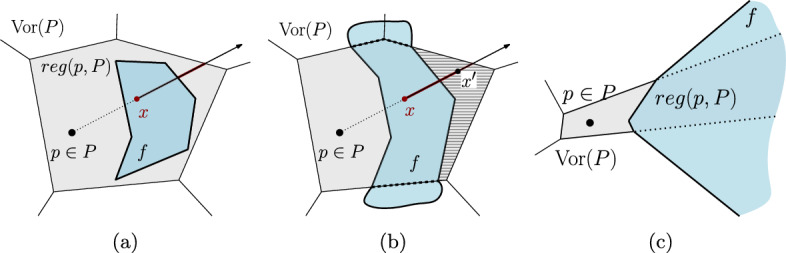
Proof of Proposition 3; the face *f* is shown in blue

Suppose first that *f* is bounded. Then $T\ne \emptyset$ because otherwise $f\subseteq \textit{reg}(p,P)$, and the visibility property of Proposition 2 would not hold for any point $x\in f$, see Fig. 5a. Suppose further that *T* is not connected, see Fig. 5b, where *T* consists of two components shown in dashed lines.

Then *f* must partition a region $\textit{reg}(p,P)$ in at least two parts, and *p* must be contained in exactly one of them, $p\in \textit{reg}(p,P)\setminus f$. Consider a point $x'$ in a face of $\textit{reg}(p,P)\setminus f$ that does not contain *p*. Then $f \cap \overline{px'}$ cannot satisfy the visibility property of Proposition 2 resulting in contradiction. Hence, *T* must be connected.

Now suppose that *f* is unbounded, see Fig. 5c. If $T\ne \emptyset$ then *T* is a forest as argued above. Suppose to the contrary that there is a tree $T_{1}$ in *T*, such that $T_{1}$ has no unbounded edge. $T_{1}$ partitions *f* into smaller faces, out of which exactly one is bounded because $T_{1}$ contains no unbounded edge; denote the unbounded face as $f_{u}$. Let $T_{u}$ be the edges of $T_{1}$ that are adjacent to the boundary of $f_{u}$. Then for any point $x\in T_{u}$, there is a sufficiently small neighborhood *N*(*x*) of *x* such that $N(x)\cap f_{u}\subset \textit{reg}(p,P)$ for a point $p\in P$. Since $p\not \in f$, then $p\in \textit{reg}(p,P){\setminus } f_{u}$. Note that $\partial f$ and *T* cross transversally. Since $f_{u}$ is unbounded and $\textit{reg}(p,P)$ is convex, it follows that $\textit{reg}(p,P)\setminus f_{u}$ has at least two connected components $C_{1},C_{2}$; without loss of generality, assume $p\in C_{1}$. Let *x* be a point in $\partial f_{u}\cap \partial C_{2}$; and let *y* be the intersection of *L*(*p*, *x*) and $\partial \textit{reg}(p,P)$. Then $y\not \in f_{u}$. By Proposition 2, the segment $\overline{xy}$ must be contained in *f*, which is a contradiction. Hence, the tree $T_{1}$ must have at least one unbounded edge incident to infinity. $\square$

The following proposition states how the cluster hull $CLH (\mathcal {P})$ characterizes the unbounded faces of the $FCVD (\mathcal {P})$.

### Proposition 4

A region ${f_{c}reg}(p,\mathcal {P})$ is unbounded if and only if *p* is a vertex of $CLH (\mathcal {P})$. A color Voronoi edge between ${f_{c}reg}(p,\mathcal {P})$ and ${f_{c}reg}(q,\mathcal {P})$, where $p\in P$ and $q\in Q\ne P$, is unbounded if and only if $\overline{pq}$ is an edge of $CLH (\mathcal {P})$.

### Proof

By definition, $p\in P$ is a hull vertex if and only if there exists a line *l* through *p* such that *P* lies entirely in one half-plane defined by *l*, denoted $H^{-}$, and every cluster $Q\ne P$ intersects the other half-plane, $H^{+}$. Consider the ray *r* from *p* that is orthogonal to *l* and is contained in $H^{+}$. Then there exists a point $p'\in r$ such that the disk with center $p'$ and radius $d(p,p')$ contains a point from every cluster $Q\ne P$ in its interior and touches *p* on its boundary. Hence, the unbounded portion of *r* starting from $p'$ is contained in ${f_{c}reg}(p,\mathcal {P})$. So the region ${f_{c}reg}(p,\mathcal {P})$ is also unbounded. Similarly, the second statement follows from the definition of hull edges. $\square$

Note that, by the above correspondence, the order of the unbounded edges of $FCVD (\mathcal {P})$ corresponds also to the angular order of the edges of the cluster hull $CLH (\mathcal {P})$. Furthermore, the normal vector associated with each hull edge is at opposite direction when considering the FCVD versus the HVD as already noted in Sect. 2.

### Proposition 5

The $FCVD (\mathcal {P})$ (as well as the $HVD (\mathcal {P})$) may have $\Theta (n\alpha (m))$ unbounded faces and edges. The bound reduces to $\Theta (n)$, if the family $\mathcal {P}$ is non-crossing or if the clusters in $\mathcal {P}$ have each cardinality $\le 2$ (i.e., $n\le 2m$).

### Proof

By Proposition 4, the unbounded faces of the $HVD (\mathcal {P})$ and the $FCVD (\mathcal {P})$ coincide, thus the same bound applies to both diagrams.

For a non-crossing family $\mathcal {P}$ the complexity of $HVD (\mathcal {P})$ has been shown to be *O*(*n*) [29], and thus, the same holds for its unbounded faces. Therefore, the unbounded faces of $FCVD (\mathcal {P})$ are also *O*(*n*) in this case, and therefore $\Theta (n)$.

Consider an arbitrary family $\mathcal {P}$. Under the well-known point-line duality transformation *T*, a point (*a*, *b*) in $\mathbb {R}^{2}$ is transformed to a non-vertical line $y=ax-b$; let the inverse transformation be denoted $T^{-1}$. For every point *p*, denote by $H^{-}(p)$ the lower half-plane below the dual line *T*(*p*).

For a cluster $P\in \mathcal {P}$, the intersection of the lower half-planes $H^{-}(P)=\bigcap _{p\in P}H^{-}(p)$ is a convex unbounded polygon, called a wedge.

Consider the arrangement of the boundaries of all the wedges $H^{-}(P)$, for $P\in \mathcal {P}$. Define *E* to be the upper envelope of this arrangement. Let *s* be a point on *E*, for some cluster $P\in \mathcal {P}$. Then all points from *P* are below (or touching) the dual line $T^{-1}(s)$. For any other cluster *Q*, there is a point $q\in Q$ such that *q* is above $T^{-1}(s)$. This shows that *E* corresponds to the unbounded cells of $FCVD (\mathcal {P})$ in directions 0 to $\pi$. Similarly, the lower envelope of the arrangement of the upper half-planes, denoted by $E'$, corresponds to the unbounded cells of $FCVD (\mathcal {P})$ in directions $\pi$ to $2\pi$. This was pointed out by Aurenhammer et al. [5] for the case of two-point clusters derived from segments. Consequently, to bound the complexity of the unbounded cells of $FCVD (\mathcal {P})$, it is enough to bound the complexity of *E* and $E'$.

We can convert any unbounded wedge $H^{-}(P)$ to a convex polygon by taking its intersection with a half-plane $\{y\ge C\}$, for a small enough constant *C*. Under this conversion *E* is a face of the common exterior of the convex polygons $H^{-}(P)$, for all $P\in \mathcal {P}$. There are *m* such polygons and their total complexity is at most *n*. As shown by Aronov et al. [3], the complexity of *E* can be $\Theta (n\alpha (m))$. Similarly, $E'$ is of the same complexity. Thus, the complexity of the unbounded cells of $FCVD (\mathcal {P})$ is $O(n\alpha (m))$ and this is tight.

If the clusters are pairs of points, then $H^{-}(P)$ is a wedge with two sides that contains the vertical ray below its apex. In this case the complexity of *E* is *O*(*n*) [5, 15], in particular, $\le 3n-2$ [28]. $\square$

Next we study the number of bounded faces of the FCVD. To capture their behavior, which may be super-linear even in the case of non-crossing clusters, we define the notion of a straddle.

### Definition 4

**Fig. 6:**
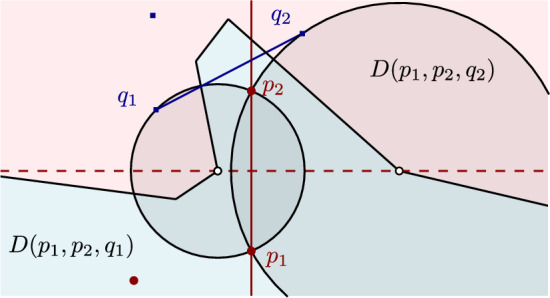
Illustration of a straddle in the $$FCVD $$ of two clusters: points $$q_1,q_2\in Q$$ straddle the pair of points $$p_1,p_2\in P$$. The hollow vertices are the centers of $$D(p_1,p_2,q_1)$$ and $$D(p_1,p_2,q_2)$$

A cluster $Q$, and in particular a pair of points $q_1,q_2 \in Q$, is said to *straddle* a pair of points $p_1,p_2 \in P$, if the disks $D(p_1,p_2,q_1)$ and $D(p_1,p_2,q_2)$ contain no points of $P$ nor $Q$ in their interior; see Fig. 6 for an example.

The term *straddle* is motivated by the necessary condition that segment $\overline{q_1q_2}$ intersects (straddles) the line $L(p_1,p_2)$, see Lemma 6. Note that if the pair $(q_1,q_2)$ straddles $(p_1,p_2)$, the segments $\overline{q_1q_2}$ and $\overline{p_1p_2}$ may or may not intersect.

### Lemma 6

If $D(p_1,p_2,q_1)$ and $D(p_1,p_2,q_2)$ contain no points of $P$ and $Q$ in their interior, then the segment $\overline{q_1q_2}$ intersects (straddles) the line $L(p_1,p_2)$.

### Proof

Suppose that the disks $D(p_1,p_2,q_1)$ and $D(p_1,p_2,q_2)$ are empty, see Fig. 6. Then, point $q_1$ lies on $C(p_1,p_2,q_1) \setminus D(p_1,p_2,q_2)$ and point $q_2$ lies on $C(p_1,p_2,q_2) \setminus D(p_1,p_2,q_1)$. Hence, line $L(p_1,p_2)$ separates them, so $\overline{q_1q_2} \cap L(p_1,p_2) \ne \emptyset$. $\square$

The centers of the two disks involved in a straddle are mixed vertices of $b_c(P, Q)$, which are incident to the Voronoi edge of bisector $b(p_1,p_2)$ in $\mathcal {V}(P)$.

### Definition 5

The number of straddles of $\mathcal {P}$ is

$$\begin{aligned}&\textit{str}(\mathcal {P}):= \sum _{P\in \mathcal {P}}\sum _{e(p_i,p_j) \in \mathcal {V}(P)}\textit{str}(p_i,p_j), \end{aligned}$$

where $\textit{str}(p_i,p_j)$ denotes the number of clusters in $\mathcal {P}$ that straddle $(p_i,p_j)$, and $e(p_i,p_j)$ denotes the Voronoi edge of $\mathcal {V}(P)$ along the bisector $b(p_i,p_j)$.

### Proposition 7

The number of straddles is $\textit{str}(\mathcal {P})\le m'(3n-5m')$, where $m'$ is the number of clusters that consist of at least two points.

### Proof

A straddle induced by a cluster $Q$ on a pair of points $(p_i,p_j)$ corresponds to a pair of vertices on $b(p_i,p_j)$ incident to the color bisector $b_c(P,Q)$, which are the centers of disks $D(p_i,p_j,q_1$) and $D(p_i,p_j,q_2)$, where $q_1,q_2\in Q$. These disks are empty of any point in $P$ or $Q$, implying that their centers lie on a Voronoi edge $e\subseteq b(p_i,p_j)$ in $\mathcal {V}(P)$. Any other disk centered on $b(p_i,p_j)$ passing through $p_i,p_j$, except $D(p_i,p_j,q_1$) and $D(p_i,p_j,q_2)$, must either contain $q_1$, or contain $q_2$, or *Q* lies entirely outside the disk and its closure. This implies that cluster *Q* may induce at most one straddle on the pair $(p_i,p_j)$ (see for example the pair $(p_1,p_2)$ and cluster $Q$ in Fig. 6). Thus, $\textit{str}(p_i,p_j) \le m'-1$ because clusters with a single point cannot straddle any pair.

The inner summation considers pair of points with a Voronoi edge in $\mathcal {V}(P)$, which is of size at most $3|P|-5$. Hence,

$$\begin{aligned} \textit{str}(\mathcal {P}) < \sum _{P\in \mathcal {P}}m'(3|P|-5)=m'(3n-5m'), \end{aligned}$$

since we only need to sum over clusters $P\in \mathcal {P}$ with at least two points. $\square$

In the following lemma, we show that certain consecutive mixed vertices along a bisector are caused by straddles, which we then use to bound the total number of mixed vertices in the $FCVD (\mathcal {P})$.

### Lemma 8

**Fig. 7:**
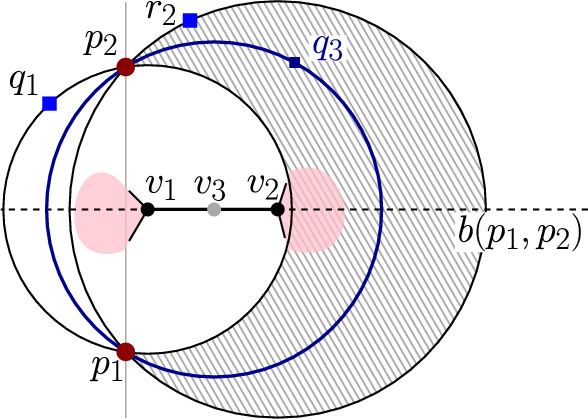
Illustration for the proof of Lemma 8

Let $v_1,v_2$ be two consecutive mixed vertices on bisector $b(p_1,p_2)$ of the $FCVD (\mathcal {P})$, where $p_1,p_2 \in P$, such that the segment $\overline{v_1v_2}$ is outside of ${f_{c}reg}(P,\mathcal {P})$,

see Fig. 7. Suppose $v_1$ is induced by cluster *Q* (i.e., $v_1$ is the center of disk $D(p_1,p_2,q_1)$, $q_1\in Q$). Then cluster *Q* straddles $(p_1,p_2)$. Analogously, we have the same for $v_2$.

### Proof

Let $q_1$ and $r_2$ be the two points that together with $p_1$ and $p_2$ induce the vertices $v_1$ and $v_2$ respectively. Without loss of generality, let $b(p_1,p_2)$ be a horizontal line and let $v_1$ be to the left of $v_2$. This implies that $q_1$ is to the left of $L(p_1,p_2)$ and $r_2$ to the right. Suppose $q_1 \in Q$. Disk $D(p_1,p_2,q_1)$ is a farthest color disk that contains no point from $P$ or $Q$ in its interior. Under the general position assumption it contains no point of $Q$, other than $q_1$, on its closure either. Thus, a (moving) disk centered on $b(p_1,p_2)$ to the right of $v_1$, passing through $p_1,p_2$, cannot be a farthest color disk, until it hits another point $q_3\in Q$ for the first time. Consider the disk $D(p_1,p_2,q_3)$ with center $v_3$. Since the disk $D(p_1,p_2,r_2)$ is a farthest color disk, it contains at least one point of *Q* within its closure, or $v_3$ lies between $v_1$ and $v_2$. Similarly, $D(p_1,p_2,q_3)$ may contain no point of *P* in its interior, and by its definition, no point of *Q*. Thus, $D(p_1,p_2,q_1)$ and $D(p_1,p_2,q_3)$ define a straddle on $b(p_1,p_2)$, i.e., *Q* straddles $(p_1,p_2)$. If $r_2\in Q$, the same straddle defines both $v_1$ and $v_2$. Otherwise, there is a straddle for $v_1$ and a different straddle for $v_2$. $\square$

**Fig. 8:**
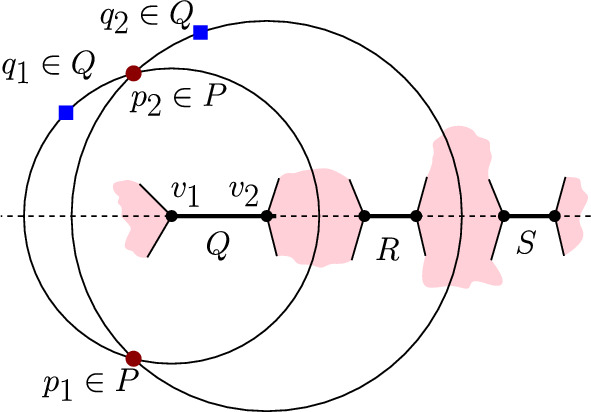
A sequence of mixed vertices on bisector $b(p_1,p_2)$ inducing multiple bounded faces of ${f_{c}reg}(P,\mathcal {P})$ (shown shaded)

### Proposition 9

$FCVD (\mathcal {P})$ has $O(n + \textit{str}(\mathcal {P}))$ bounded faces.

### Proof

By Proposition 3, each bounded face is incident to at least two distinct mixed vertices that are the leaves of its internal skeleton. Each mixed vertex is incident to two faces. Thus, the number of bounded faces is bounded from above by the total number of mixed vertices.

For any cluster $P\in \mathcal {P}$, and for any pair of points $(p_i,p_j)$ inducing an edge *e* in $\mathcal {V}(P)$, we count the number of mixed vertices appearing along *e*. By Lemma 8, only the two outermost mixed vertices on *e* may not be the result of a straddle; any other pair of consecutive mixed vertices corresponds to a straddle. Thus, there are at most $2\cdot \textit{str}(p_i,p_j) +2$ mixed vertices incident to *e*, see Fig. 8. Summing up over all Voronoi edges in $\mathcal {V}(P)$, for all $P\in \mathcal {P}$, the total number of possible mixed vertices is $O(n + \textit{str}(\mathcal {P}))$. $\square$

By combining Propositions 5 and 9, Euler’s formula for planar graphs, and the fact that Voronoi vertices have degree at least 3, we conclude the following.

### Theorem 1

The $FCVD (\mathcal {P})$ has combinatorial complexity $O(n\alpha (m) + \textit{str}(\mathcal {P}))$, where $\textit{str}(\mathcal {P}))\le m(3n-5m)$. If $\mathcal {P}$ is non-crossing, or it consists of point-pairs, the complexity is $O(n+ \textit{str}(\mathcal {P}))$.

The above theorem refines the *O*(*mn*) upper bound of [1, 18], and gives a sufficient condition under which the $FCVD (\mathcal {P})$ has near-linear complexity. The term $O(n\alpha (m))$ gives a tight upper bound^1^ to the unbounded faces, while the term $O(n+ \textit{str}(\mathcal {P}))$ to the bounded ones. Straddles capture the main reason behind the possible superlinear complexity of the FCVD, which is due to multiple bounded faces of the same cluster that are empty of inner Voronoi vertices. Due to straddles, the FCVD may have a quadratic number of bounded faces, even if $\mathcal {P}$ is non-crossing, as it will be shown in Sect. 5. This is in contrast to the $HVD (\mathcal {P})$, which has complexity *O*(*n*), if the family $\mathcal {P}$ is non-crossing.

### Corollary 10

If $\mathcal {P}$ is non-crossing and $\textit{str}(\mathcal {P})=O(n)$, the $FCVD (\mathcal {P})$ has complexity *O*(*n*).

## Relation to Abstract Voronoi Diagrams

In this section, we consider the FCVD under the framework of abstract Voronoi diagrams. If the family of clusters $\mathcal {P}$ satisfies the axioms of the framework, then the FCVD is a simple tree of linear combinatorial complexity.

Abstract Voronoi diagrams were introduced by Klein [21]. They are defined in terms of bisecting curves that satisfy some simple combinatorial properties, called axioms, and they offer a unifying framework to various concrete Voronoi instances. In the context of color Voronoi diagrams on a family of clusters $\mathcal {P}$, these axioms are interpreted as follows.

For every subset $\mathcal {P}' \subseteq \mathcal {P}$:

1. Each region ${n_{c}reg}(P, \mathcal {P}')$ is non-empty and connected.
2. Each point in the plane belongs to the closure of a region ${n_{c}reg}(P, \mathcal {P}')$.
3. Each color bisector $b_c(P,Q)$, where $P,Q\in \mathcal {P}$, is an unbounded simple curve.

If the underlying system of color bisectors satisfies axioms (A1)-(A3), the family of clusters $\mathcal {P}$ is called *admissible*. Then the $FCVD (\mathcal {P})$ is a concrete instance of the farthest abstract Voronoi diagram [9, 24] on a set of *m* sites (clusters), whose bisectors, however, have non-constant complexity; one color bisector may have complexity $\Theta (n)$.

### Proposition 11

If $\mathcal {P}$ is admissible, then the $FCVD (\mathcal {P})$ is a tree of complexity *O*(*n*).

### Proof

Since $\mathcal {P}$ is admissible, $FCVD (\mathcal {P})$ is an instance of the farthest abstract Voronoi diagram. By [24], it is a tree consisting of *O*(*m*) (unbounded) faces, thus, its number of pure Voronoi vertices is *O*(*m*). It remains to bound the mixed Voronoi vertices.

Let *f* be a face of ${f_{c}reg}(P,\mathcal {P})$ and consider $T=\mathcal {V}(P)\cap f$. If $T=\emptyset$, then there is no mixed Voronoi vertex on $\partial f$; otherwise, *T* is a forest (by Proposition 3(2)). The trees in *T* satisfy the following properties: each tree has an unbounded edge; the remaining leaves are mixed vertices on $\partial f$; other vertices are internal vertices of degree 3. In a tree where all vertices are of degree 1 or 3, the number of leaves is exactly 2 plus the number of degree-3 vertices. Let $I_{f}$ be the number of internal vertices of *T*, then the number of mixed vertices on $\partial f$ is at most $I_{f}+2$. Every internal vertex is contained in exactly one face *f*, and the total number of internal vertices is *O*(*n*). Since every mixed Voronoi vertex appears on the boundary of some face *f*, then, summing over all faces, we conclude that the total number of mixed Voronoi vertices is *O*(*n*). $\square$

**Fig. 9:**
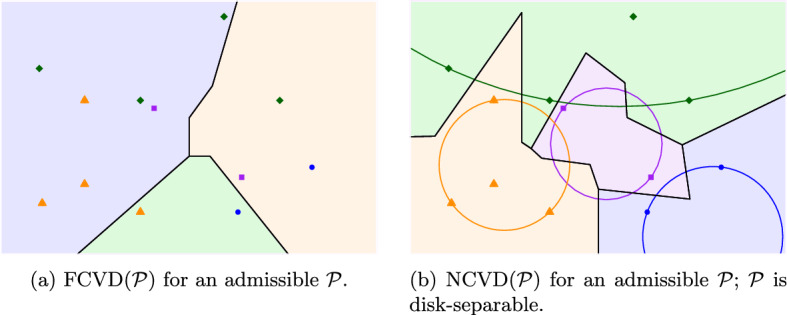
Color Voronoi diagrams of an admissible family of four clusters

Note that the regions of $FCVD (\mathcal {P})$ need not be connected. Figure 9 illustrates a farthest (a) and a nearest (b) color Voronoi diagram for an admissible bisector system.

Next, we give a necessary and sufficient condition under which a family of clusters is admissible.

### Proposition 12

$\mathcal {P}$ is admissible if and only if the following conditions hold:

1. $\mathcal {P}$ is non-enclosing.
2. All regions of $NCVD (\mathcal {P})$ are connected.

### Proof

If $\mathcal {P}$ is admissible, then condition (2) holds by axiom (A1). Further, by (A3), all bisectors are unbounded, thus, condition (1) also holds, by Lemma 1.

Conversely, suppose $\mathcal {P}$ satisfies conditions (1) and (2). Given a set of points $P$, their Voronoi regions can only shrink if we add a new site. As a result, ${n_{c}reg}(P,\mathcal {P}) \subseteq {n_{c}reg}(P,\mathcal {P}')$, for any family of clusters $\mathcal {P}' \subset \mathcal {P}$. Furthermore, any face of ${n_{c}reg}(P,\mathcal {P}')$ must contain some distinct point of $P$; thus, no face of ${n_{c}reg}(P,\mathcal {P}')$ can become empty in ${n_{c}reg}(P,\mathcal {P})$. Then by condition (2), no region of ${n_{c}reg}(P,\mathcal {P}')$ can be disconnected, because otherwise, ${n_{c}reg}(P,\mathcal {P})$ would also remain disconnected. Thus, the connectivity requirement of axiom (A1) is satisfied. Furthermore, no pair of clusters can have a color bisector that consists of more than one connected component. Then, assuming condition (1), each color bisector must consist of a single unbounded branch, which is a simple curve satisfying axiom (A3). The remaining axioms are trivially satisfied. $\square$

It is worth to note that, unlike the HVD [29], a family of clusters $\mathcal {P}$ which is non-crossing, and even linearly separable, need not be admissible. For example, Fig. 10 illustrates the bisectors of three linearly separable clusters $b_c(P,Q)$ and $b_c(Q,R)$, which may intersect $\Theta (|P|+|Q|+|R|)$ times, which violates axiom (A1). However, if the input family $\mathcal {P}$ is linearly separable or simply non-crossing, then we can determine if $\mathcal {P}$ is admissible by checking whether the regions of $NCVD (\mathcal {P})$ are indeed connected or not (by Proposition 12).

### Corollary 13

If $\mathcal {P}$ is non-enclosing, then we can decide if $\mathcal {P}$ is admissible in $O(n\log n)$ time.

**Fig. 10:**
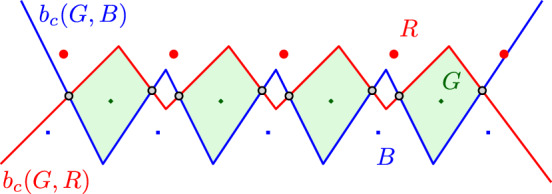
Three linearly separable clusters *R*, *G*,  and *B*, where bisectors $b_c(G,B)$ and $b_c(G,R)$ intersect $\Theta (n)$ times. The (shaded) region ${n_{c}reg}(G, \{R,G,B\})$ has $\Theta (n)$ faces

Next we define *disk-separability* and show that it gives a sufficient condition for a set of clusters to be admissible. A family of clusters $\mathcal {P}$ is called *disk-separable* if, for every cluster $P\in \mathcal {P}$, there exists a disk, which contains $P$ but does not contain any point from any other cluster $Q\in \mathcal {P}\setminus \{P\}$. Refer to Fig. 9b for an example of disk-separable clusters.

### Theorem 2

If $\mathcal {P}$ is disk-separable, then $\mathcal {P}$ is admissible; thus, $FCVD (\mathcal {P})$ is a tree of complexity *O*(*n*).

### Proof

Disk-separability clearly implies linear separability, thus, axiom (A3) is satisfied. We only need to show that ${n_{c}reg}(P,\mathcal {P})$ is connected, for any $P\in \mathcal {P}$.

Consider a cluster $P\in \mathcal {P}$ and let *D* be a disk, centered at some point *o*, that contains $P$ but does not contain any point from any other cluster, see Fig. 11. Consider the line segment $\overline{op}$ for a point $p \in P$. For any point $x \in \overline{op}$ and $q \notin D$, the distance $d(x,p)<d(x,q)$, as the circle centered at *x* passing through *p* is fully contained in *D*, while *q* lies outside *D*. Thus, segment $\overline{op}$ is entirely contained in ${n_{c}reg}(P,\mathcal {P})$, as no point from any other cluster is contained in *D*. Since any component of ${n_{c}reg}(P,\mathcal {P})$ must contain some point $p\in P$, and thus, it must also contain $\overline{op}$, it follows that ${n_{c}reg}(P,\mathcal {P})$ is connected. $\square$

**Fig. 11:**
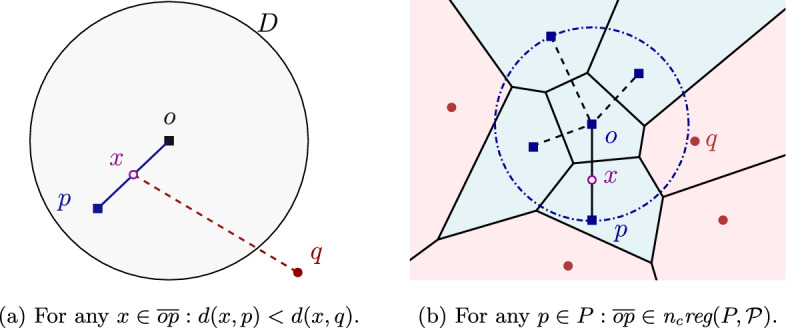
Illustrations for the proof of Proposition 2

In the next section, we show that linear separability is not sufficient to guarantee linear complexity for the FCVD.

## A lower bound for linearly separable clusters

In this section we show that the FCVD may have quadratic complexity, even if the clusters are linearly separable. To this aim, we present a construction of a family of non-crossing point-pairs $\mathcal {P}$ such that $$\textit{str}(\mathcal {P})=\left( {\begin{array}{c}m\\ 2\end{array}}\right) $$ and the straddles all result in mixed Voronoi vertices. At the end, a small perturbation can transform the set $\mathcal {P}$ into being linearly separable.

We define a family $\mathcal {P}$ of *m* non-crossing clusters of size 2 ($n=2m$), such that $FCVD (\mathcal {P})$ contains $\Omega (m^{2})$ mixed Voronoi vertices. Let

$$\begin{aligned}&\mathcal {P}=\{P_i = \{ l_i,u_i\}, 1\le i\le m\}. \end{aligned}$$

**Fig. 12:**
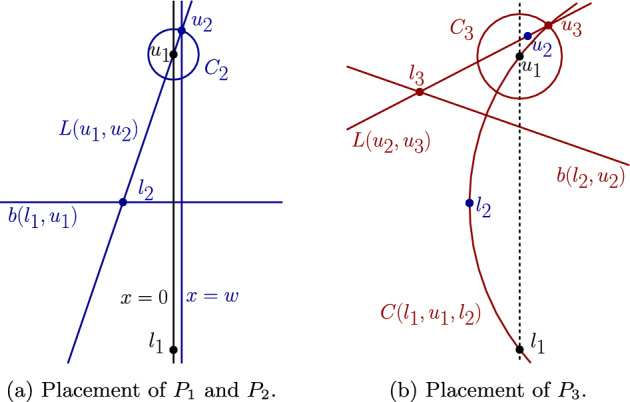
Construction of the set $\mathcal {P}$, for $m=3$

Let $l_1=(0,0)$ and $u_1=(0,2^m)$. Let $C_i$, $2 \le i \le m$, be a set of concentric circles centered at $u_1$, each of radius $2^{-m+i-2}$. We control the placement of all other points using a parameter *w*, with $0 \le w \ll 2^{-m}$. In particular, point $u_2$ is placed at the upper intersection point of circle $C_2$ and the vertical line $x=w$. Each lower point $l_i$, for $i\ge 2$, is placed at the intersection point of line $L(u_{i-1},u_{i})$ and bisector $b(l_{i-1},u_{i-1})$. Each upper point $u_i$, for $i\ge 3$, is placed on the upper intersection of circles $C_i$ and $C(l_{i-2},u_{i-2},l_{i-1})$. Refer to Fig. 12 for $m=3$. The idea behind the construction is that the pair $(l_i,u_i)$ straddles $(l_{i-1},u_{i-1})$ when *w* is small.

The construction of $\mathcal {P}$ is summarized below. Let $C_a \cap _{up}C_b$ denote the intersection point of $C_a\cap C_b$ with the largest *y* coordinate.

$$\begin{aligned} l_i =&{\left\{ \begin{array}{ll} (0,0), &  \text {if } i=1\\ L(u_{i-1},u_{i}) \cap b(l_{i-1},u_{i-1}),& \text {if } i\ge 2 \end{array}\right. } \ \ \ \\ u_i =&{\left\{ \begin{array}{ll} (0,2^m), &  \text {if } i=1\\ C_i \cap _{up}(x = w),&  \text {if } i=2\\ C_i \cap _{up}C(l_{i-2},u_{i-2},l_{i-1}),&  \text {if } i\ge 3 \end{array}\right. }&\end{aligned}$$

The construction for various values of *w* and *m* can be visualized interactively on a Geogebra applet^2^. Let *x*(*p*) and *y*(*p*) denote the *x*- and *y*-coordinate of a point *p* respectively. The quantity *w* needs to be sufficiently small so that all clusters can be appropriately placed.

Cluster $(l_i,u_i), i\ge 2,$ is said to be *correctly placed* if $y(u_i)>y(u_{i-1})$, and $y(u_1)> y(l_i)>y(l_{i-1})$.

### Lemma 14

If $w=0$, then all points from $\mathcal {P}$ lie on the *y*-axis, with their *y* coordinates ordered as follows: $y(l_i)< y(l_j)< y(u_i) < y(u_j)$, for $i<j$.

### Proof

If $w=0$, then $x(u_2)=0$. By induction, every line $L(u_{i-1},u_i)$ and every circle $C(l_{i-2},u_{i-2},l_{i-1})$ degenerate to the line $x=0$. For any $i\ge 2$, the coordinates of the points can be shown to be the following by induction:

1 $$\begin{aligned} l_i&= (0,2^m - 2^{m-i+1} +2^{i-2-m}/3 - 2^{2-i-m}/ 3), \end{aligned}$$

2 $$\begin{aligned} u_i&= (0,2^m+2^{i-2-m}). \end{aligned}$$

The claimed ordering of the *y* coordinates follows. $\square$

Let $\theta _1(w)$ be the angle between the *y*-axis and $L(u_1,u_2)$. For $j\ge 2$, let $\theta _j(w)$ be the angle between $L(u_{j-1},u_{j})$ and $L(u_j,u_{j+1})$. Note that all angles $\theta _i (w)$’s are functions of *w*. For brevity, we write $\theta _{i}=\theta _{i}(w)$, when there is no ambiguity. We proceed to show properties of the angles when *w* is sufficiently small.

### Lemma 15

For all $1\le j\le m$, it holds: $\lim _{w\rightarrow 0} \theta _j(w)=0$.

### Proof

We prove by induction. The base case when $j=1$ holds because $\theta _1(w)=\arcsin (2^{m} w)$. For the inductive step, we show that $\lim _{\theta _{ k-1}\rightarrow 0}\theta _k=0$ for $k\ge 2$. Then the lemma follows.

**Fig. 13:**
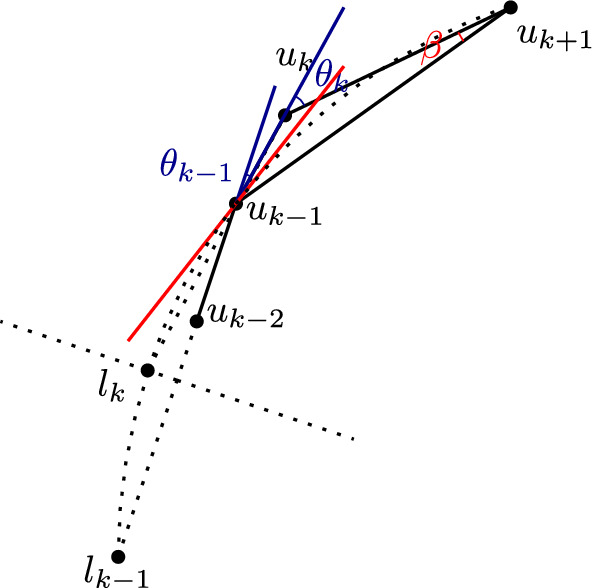
Proof of Lemma 15

Refer to Fig. 13, let $\theta _k=\alpha +\beta$, where $\alpha =\angle u_ku_{k-1}u_{k+1}$ and $\beta =\angle u_ku_{k+1}u_{k-1}$. Consider the tangent line (red) at $u_{k-1}$ to $C(l_{k-1},l_{k},u_{k-1})$ which splits $\alpha$ into two angles. By elementary geometry, we get $\alpha =\arcsin \left( \frac{d(l_k,u_{k-1})}{2r}\right) +\arcsin \left( \frac{d(u_{k-1},u_{k+1})}{2r}\right) ,$ where *r* is the radius of $C(l_k,l_{k-1},u_{k-1})$.

Note that $\lim _{\theta _{k-1}\rightarrow 0}r=\infty$, and $d(l_k,u_{k-1})$, $d(u_{k-1},u_{k+1})$ are at most a constant. Then $\lim _{\theta _{k-1}\rightarrow 0}\alpha =0$.

Since $\frac{d(u_k,u_{k-1})}{\sin \beta }=\frac{d(u_k,u_{k+1})}{\sin \alpha }$ and $\frac{d(u_k,u_{k-1})}{d(u_k,u_{k+1})}\le \frac{3}{2}$, then $\sin \beta \le \frac{3}{2}\sin \alpha$. Therefore, $\lim _{\theta _{k-1}\rightarrow 0}\beta =0$.

Hence, we have

$$\begin{aligned} \lim _{\theta _{k-1}\rightarrow 0}\theta _k=\lim _{\theta _{k-1}\rightarrow 0}\alpha +\beta =0. \end{aligned}$$

$$\square $$

### Lemma 16

If $w>0$ is sufficiently small, then the coordinates of any point from $\mathcal {P}$ are sufficiently close to the coordinates of the same point when $w=0$. Consequently, the clusters $P_1,\cdots ,P_m$ are correctly placed.

### Proof

By Lemma 15, $\lim _{w\rightarrow 0} \theta _j(w)=0$. By definition of the angles, all points $u_i, l_{i}$’s are sufficiently close to their positions when $w=0$. By Lemma 14, the clusters are correctly placed when $w=0$. Hence they are also correctly placed for sufficiently small *w*. $\square$

### Lemma 17

If $w>0$ is sufficiently small, for any $k\ge 2$, the followings hold:

1. $$1.5\theta _{k-1}\le \theta _{k}$$,
2. $$\sum _{i=1}^{k-1}\theta _i \le 2\theta _k$$.

### Proof

The setup of this proof is the same as that of Lemma 15 (refer to Fig. 13). We have $\theta _{k}=\alpha +\beta$ and $\alpha > \theta _{k-1}$. By Lemma 16, the coordinates of $u_i$’s are sufficiently close to their coordinates when $w=0$. Hence, $d(u_k,u_{k+1})\approx 2d(u_{k-1},u_k)$ by Equation (2). Thus, $\beta \approx \alpha /2$. Therefore, $\theta _k=\alpha +\beta \ge 1.5\theta _{k-1}$.

Part (2) follows from part (1) by induction on *k*, as follows. The base case, $k=2$, is immediate. For the inductive step, assume it holds for $\theta _{k-1}$ and $k\ge 3$. Then we have $\sum _{i=1}^{k-1}\theta _{i} = \sum _{i=1}^{k-2}\theta _{i}+\theta _{k-1} \le 2\theta _{k-1}+\theta _{k-1} = 3\theta _{k-1} \le 2\theta _{k}$ (where the last step follows from (1)). $\square$

### Corollary 18

If $w>0$ is sufficiently small, then the $u_i$’s form a convex chain, that is, $u_k$ is on the right-hand side of $L(u_i,u_j)$ for any $1\le i<j<k\le m$.

### Proof

By Lemma 17, we know that $u_{k+1}$ is on the right-hand side of $L(u_{k-1},u_k)$ as $\theta _k\ge \theta _1=\arcsin (2^{m} w)> 0$. The lemma follows by induction. $\square$

**Fig. 14:**
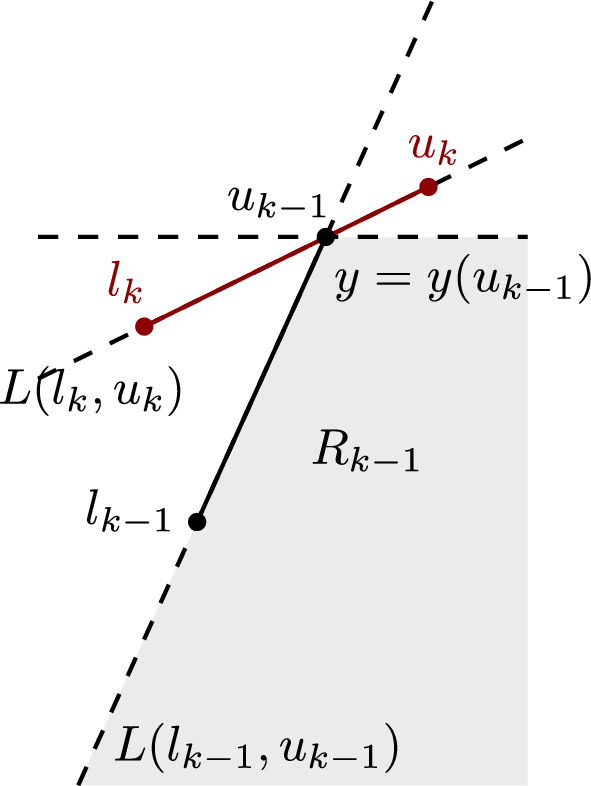
Proof of Lemma 19

### Lemma 19

If $w>0$ is sufficiently small, then for all $1\le i<j\le m$, the followings hold:

1. Points $l_i$ and $u_i$ are on the right-hand side of $L(u_j,l_j)$ for $i<j$.
2. Point $l_j$ is on the left of $L(u_i,l_i)$ and point $u_j$ is on the right.

### Proof

If $w>0$ is sufficiently small, then the clusters $P_1,\cdots ,P_m$ are correctly placed, by Lemma 16. By construction, $l_{i},u_{i}$ are on the right-hand side of $L(l_{i+1},u_{i+1})$. Let $R_i$ be the intersection of the right half-plane of $L(u_{i},l_{i})$ and $\{(x,y):y\le y(u_{i})\}$, see Fig. 14. Then $\{u_i,l_i\}$ is contained in $R_{i+1}$. Since the clusters are correctly placed, then $R_i\subseteq R_{i+1}$. Hence, $\{u_i,l_i\}\subseteq R_{i+1}\subseteq R_j$, (1) follows.

By construction, $u_j$ is on the right-hand side of $L(u_i,l_i)$. Note that $u_j,l_j$ are not contained in $R_i$. Since $y(l_j)<y(u_i)$, then $l_j$ must be on the left of $L(l_i,u_i)$. This shows (2). $\square$

### Corollary 20

For all $1\le i<j\le m$, cluster $(u_{j},l_{j})$ straddles $(u_{i},l_{i})$.

### Proof

It is enough to show that $D(u_{i},l_{i},u_{j})$ is empty of $l_{j}$ and $D(u_{i},l_{i},l_{j})$ is empty of $u_{j}$. By Lemma 19, $u_{j}$ is above $L(l_{j},u_{i})$ is on the right of $L(u_{i},l_{i})$, therefore $u_{j}$ is not in $D(u_{i},l_{i},l_{j})$. Similarly, $D(u_{i},l_{i},u_{j})$ is empty of $l_{j}$. $\square$

In fact, it is easy to show that cluster $u_{i},l_{i}$ does not straddle $(u_{j},l_{j})$ because $u_{i}\in D(l_{j},u_{j},l_{i})$, for $1\le i<j\le m$. Since each cluster consists of only two points, the straddles described in Corollary 20 are all the straddles in the example. Hence, there are exactly $$\left( {\begin{array}{c}m\\ 2\end{array}}\right) $$ straddles in $\mathcal {P}$.

### Lemma 21

If $w>0$ is sufficiently small, then $u_j$ lies to the right of $L(l_k,u_i)$, for any $i<k<j$.

**Fig. 15:**
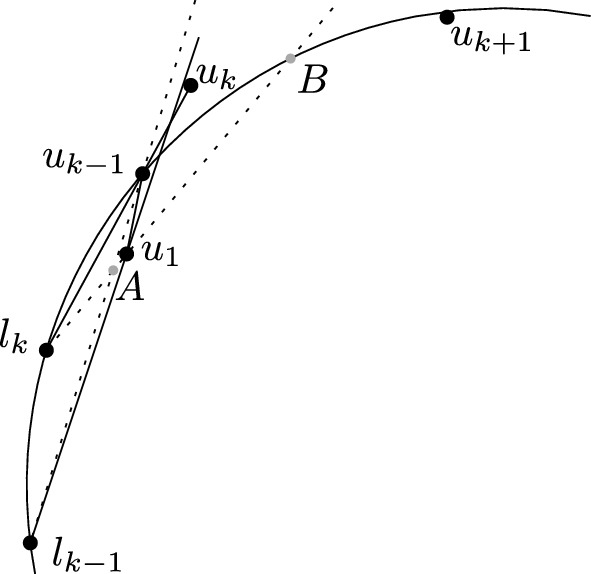
Proof of Lemma 21

### Proof

By Lemma 18, it suffices to show that $u_{k+1}$ is on the right-hand side of $L(l_k,u_1)$. Let $A=L(l_k,u_1)\cap L(l_{k-1},u_{k-1})$, and let $B=L(l_k,u_1)\cap _{up}C(l_{k-1},l_k,u_{k-1})$, see Fig. 15. Then $\angle u_1Au_{k-1}> \angle l_ku_{k-1}u_{k-2}=\theta _{k-1}$, where the equality follows from the definition of $\theta _{k-1}$ and the collinearity of $l_{k}, u_{k-1}, u_{k}$.

If *w* is sufficiently small, by Lemma 16, the positions of points are close to their positions when $w=0$. Then by Equation (2), we have $d(u_1,u_{k-2})\approx d(u_{k-2},u_{k-1})$. Thus, $\angle Au_{k-1}u_1\approx \angle u_{k-2}u_1u_{k-1}< \sum _{i=1}^{k-2}\theta _i/2\le \theta _{k-1}$, where the last inequality follows from Lemma 17(2). Hence, $\angle u_1Au_{k-1}\ge \angle Au_{k-1}u_1$, so $d(u_{k-1},u_1)\ge d(A,u_1)$.

Suppose that the lemma were incorrect, that is, $u_{k+1}$ lies on the left-hand side of $L(l_k,u_1)$. Then $d(A,B)/d(A,u_{k-1})\ge 5/2$, while $d(A,l_{k-1})/d(l_k,A)\approx 2$. However, these two ratios should be equal by the intersecting chords theorem, which is a contradiction. Thus, the lemma holds. $\square$

Finally, we show that the set of disks $D(u_i,l_i,u_j)$, with $i<j$, are all farthest color disks. Note that each such disk corresponds to a straddle. To this aim we show the following.

### Lemma 22

If $w>0$ is sufficiently small, then $P_k\cap D(u_i,l_i,u_j)\ne \emptyset$, for $i<j$ and all $k\ne i,j$.

### Proof

There are three cases: $k<i$, $i<k<j$, and $k>j$, which we prove separately.

Suppose $k<i$, we show $u_k\in D(u_i,l_i,u_j)$. By Lemmas 18 and 19(2), $u_k$ is below $L(u_i,u_j)$ and on the right of $L(l_i,u_i)$. By construction, $u_k$ is above $L(l_i,u_j)$. Thus, $u_k$ lies in the triangle formed by $l_i,u_i,u_j$. Hence, $u_k\in D(u_i,l_i,u_j)$.

Suppose $i<k<j$, we show $l_k\in D(u_i,l_i,u_j)$. By Lemma 19(2), $l_k$ is on the left of $L(u_i,l_i)$ while $u_j$ is on the right. Thus, if $r(D(u_i,l_i,u_j))<r(D(u_i,l_i,l_k))$, where $r(\cdot )$ denotes the radius, then $D(u_i,l_i,u_j)$ contains the part of $D(u_i,l_i,l_k)$ that is on the left of $L(u_i,u_j)$ which contains $l_k$. It remains to show that there exists *w* such that $r(D(u_i,l_i,u_j))<r(D(u_i,l_i,l_k))$. When *w* is small enough, the comparison of the radii of these two disks is equivalent to the comparison of the slopes of bisectors $b(l_k,u_i)$ and $b(u_j,u_i)$. Both slopes are negative, and by Lemma 21, the slope of $b(l_k,u_i)$ is larger than the slope of $b(u_j,u_i)$ when *w* is small enough. Consequently, the radius of disk $D(u_i,l_i,l_k)$ is larger than the radius of disk $D(u_i,l_i,u_j)$.

Suppose $k>j$, we show $u_k\in D(u_i,l_i,u_j)$. By Lemma 19(2), both $u_j$ and $u_k$ lie on the right-hand side of $L(u_i,l_i)$, so it suffices to show $r(D(u_i,l_i,u_j))>r(D(u_i,l_i,u_k))$. Again, comparing the two radii is equivalent to comparing the slopes of bisectors $b(u_i,u_j)$ and $b(u_i,u_k)$. Result follows from Lemma 18. $\square$

There are in total $$\left( {\begin{array}{c}m\\ 2\end{array}}\right) $$ such disks and the centers of these disks are all mixed vertices of the FCVD. Hence, the FCVD of our construction has $\Omega (m^{2})$ vertices.

As mentioned in the beginning of this section, the clusters in our construction are not linearly separable because $l_i,u_{i-1},u_i$ are collinear. Furthermore, the points are not in general position because every four points $(l_{i-2},u_{i-2},l_{i-1},u_i)$, for $3\le i\le m$ are co-circular. We can easily fix these two issues by translating $l_i$ to the left infinitesimally (by an amount of $\epsilon$ with $\epsilon \ll w$) at each step of the construction so that the clusters are linearly separable and in general position while all other relations are preserved. As a result, and combining with the trivial $\Omega (n)$ lower bound, we conclude the following:

### Theorem 3

Given a linearly separable set of clusters $\mathcal {P}$ with *m* clusters and *n* points, $FCVD (\mathcal {P})$ may have $\Omega (n + m^{2})$ combinatorial complexity in the worst case.

## Construction algorithms

We compute $FCVD (\mathcal {P})$ using the standard divide and conquer paradigm: split $\mathcal {P}$ in two sets $\mathcal {P}_A$ and $\mathcal {P}_B$ of roughly equal size; recursively compute $FCVD ^{*}(\mathcal {P}_A)$ and $FCVD ^{*}(\mathcal {P}_B)$; merge the two diagrams to obtain $FCVD ^{*}(\mathcal {P})$. The merging process amounts to computing a *merge curve* *M*, which is the portion of $FCVD (\mathcal {P}_A \, \cup \, \mathcal {P}_B)$ bounding the regions of clusters in $\mathcal {P}_A$ and $\mathcal {P}_B$.

Computing the merge curve *M* consists of two main tasks.

1. Computing a starting point for each connected component of *M*;
2. Tracing each connected component of *M* through $FCVD ^{*}(\mathcal {P}_A)$ and $FCVD ^{*}(\mathcal {P}_B)$.

To perform task (2) efficiently, we further refine the diagrams $FCVD ^{*}(\mathcal {P}_A)$ and $FCVD ^{*}(\mathcal {P}_B)$ by the *visibility-based decomposition*, which was first defined for the HVD [29]. The visibility-based decomposition is a refinement of the diagram using Proposition 2: for each region ${f_{c}reg}(p,\mathcal {P}_x)$, $x=A,B$, and for each vertex *u* on $\partial {f_{c}reg}(p,\mathcal {P}_x)$, draw $L(p,u)\cap {f_{c}reg}(p,\mathcal {P}_x)$. Given this refinement, the tracing of the merge curve *M* can be performed in time linear on the complexity of $FCVD (\mathcal {P}_A \, \cup \, \mathcal {P}_B)$ using standard techniques, see e.g., [29].

To perform task (1), we first need to identify starting points on the unbounded components of *M*. These can be identified in linear time by merging the cluster hulls $CLH (\mathcal {P}_A)$ and $CLH (\mathcal {P}_B)$ to compute $CLH (\mathcal {P}_A\cup \mathcal {P}_B)$, similarly to the HVD [29]. Recall that the unbounded portions of the HVD and FCVD coincide. Thus, if the merge curve consists of only unbounded curves, we can compute them in time $O(|CLH (\mathcal {P}_A)| +|CLH (\mathcal {P}_B|))$. If $\mathcal {P}$ is admissible, we obtain the following result.

### Theorem 4

Given an admissible family of point clusters $\mathcal {P}$, the diagram $FCVD (\mathcal {P})$ can be constructed in $O(n\log n)$ time.

Let us now study an arbitrary family $\mathcal {P}$, which need not be admissible. To identify starting points on each bounded component of the merge curve, we perform searches on the internal edges of $FCVD ^{*}(\mathcal {P}_A)$ and $FCVD ^{*}(\mathcal {P}_B)$ to identify any mixed vertices incident to the merge curve. Proposition 3(1) assures that such mixed vertices exist for any bounded component of *M*. To perform the search efficiently we will use the *intersection search data structure* of Iacono et al. [19], which allows to preprocess two plane straight-line graphs *R* and *B*, so that for each edge *e* of *R*, all intersections of *e* with *B* are implicitly stored in the order they appear along *e*. The intersections are represented with a balanced binary search tree $T_e$ that can be navigated in constant time per operation.

We first need the following lemma on color bisectors, so that we can set up the navigation process in the data structure. The navigation process is a set of rules on how to navigate the data structure in order to identify mixed vertices.

### Lemma 23

Let $e\in \mathcal {V}(P)$ be a Voronoi edge between $p_{1},p_{2}\in P$; let $Q$ be a different cluster. Edge *e* intersects the color bisector $b_c(P,Q)$ at most twice. In particular, they intersect twice if and only if $Q$ straddles $(p_{1},p_{2})$; in this case, the middle portion of *e*, between the two intersection points, is farther from *Q* than from *P*.

### Proof

An intersection point of *e* with $b_c(P,Q)$ is the center of a disk empty of $P\cup Q$. By the same argument as in the proof of Proposition 7, there are at most two such disks, hence at most two intersection points. If there are two intersection points, then *Q* straddles $(p_{1},p_{2})$, by the definition of a straddle. The remaining claim follows from a similar argument as in the proof of Lemma 8. $\square$

We give the algorithms to compute the starting points on the bounded components of *M* in the following lemma.

### Lemma 24

We can compute starting points for all the bounded components of the merge curve *M* in $O((n+\textit{str}(\mathcal {P}))\log ^{2} n)$ time.

### Proof

We perform a search on all the internal edges of $FCVD ^{*}(\mathcal {P}_A)$ and $FCVD ^{*}(\mathcal {P}_B)$. Let $e \in {f_{c}reg}(P,\mathcal {P}_A)$ be an internal edge of $FCVD ^{*}(\mathcal {P}_A)$, for cluster $P\in \mathcal {P}_{A}$. The goal is to identify all portions of *e* which are farther from $P$ than from any cluster in $\mathcal {P}_{B}$, or report that no such portion exists. The endpoints of these portions are mixed vertices of the $FCVD ^{*}(\mathcal {P}_A\cup \mathcal {P}_{B})$ and they are incident to the merge curve.

To perform the search efficiently, we use the $\text {intersection search data structure}$ of Iacono et al. [19] to construct a balanced binary search tree $T_{e}$, whose nodes are intersection points of *e* with edges of $FCVD ^{*}(\mathcal {P}_B)$. We navigate on *e*, using $T_{e}$, as follows. Assume that $e=\overline{uv}$ bisects points $p_{1},p_{2}\in P$; let *x* be an intersection between *e* and $e'\in FCVD ^{*}(\mathcal {P}_B)$; there are two cases: $e'$ is an internal edge or $e'$ is a color bisector edge. The navigation process is the same in both cases, thus, we only present the former.

Suppose $e'$ is an internal edge that bisects $q_{1},q_{2}\in Q$ for a cluster $Q\in \mathcal {P}_{B}$. We compare the distances of the points *x*, *u*, *v* to clusters *P*, *Q* and do the following.

1. If $d_c(x,P)>d_c(x,Q)$ then *x* is farthest from cluster $P$ among all clusters in $\mathcal {P}$, so it must lie on an internal edge of the merged diagram $FCVD ^{*}(\mathcal {P}_{A}\cup \mathcal {P}_{B})$. If either $\overline{xu}$ or $\overline{xv}$ is entirely contained in a component of *M*, then the component of *M* contains an endpoint *u* or *v*. Such components can be found in a total of $O(n\log n)$ time using the visibility-based decomposition in a similar way as in [29][Lemma 11]. Otherwise, one traverses along *e*, starting from *x*, and finds the endpoint of the sub-segment contained in the component of *M* in $O(\log n)$ time using point-location query in $FCVD ^{*}(\mathcal {P}_{B})$.
2. If $d_c(x,P)<d_c(x,Q)$ then *x* is not on an internal edge of cluster $P$ in $FCVD ^{*}(\mathcal {P}_{A}\cup \mathcal {P}_{B})$. There are three cases.
  - If $d_c(u,P)<d_c(u,Q)$ and $d_c(v,P)<d_c(v,Q)$ then $b_c(P,Q)$ intersects *e* either 0 or 2 times. If they intersect twice, by Lemma 23, then the middle portion of *e* is farther from $Q$ than from $P$, which is a contradiction. Hence, they do not intersect, and the whole edge *e* is farther from $Q$ than from $P$, so we stop searching on *e*.
  - Suppose $d_c(u,P)<d_c(u,Q)$ and $d_c(v,P)>d_c(v,Q)$. By Lemma 23, there is exactly one intersection point between $b_c(P,Q)$ and *e* that lies on $\overline{vx}$. So we navigate to the child of *x* that is closer to *v*.
  - Suppose $d_c(u,P)>d_c(u,Q)$ and $d_c(v,P)>d_c(v,Q)$. By Lemma 23, there are exactly two intersections between $b_c(P,Q)$ and *e*, lying on $\overline{ux},\overline{vx}$ respectively. This corresponds to a straddle. In this case, we need to navigate to both children of the node *x*.

In the case where $e'$ is part of a color bisector that bisects $Q_{1},Q_{2} \in \mathcal {P}_{B}$, we compare the distances from points *u*, *v*, *x* to clusters $P,Q_{1},Q_{2}$. The navigation process remains the same.

The correctness of the navigation process follows from the fact that every bounded component of *M* encloses at least one bounded face of $FCVD (\mathcal {P}_{A}\cup \mathcal {P}_{B})$. Thus, by Proposition 3(1), a bounded component of *M* encloses at least one internal edge *e* of $FCVD ^{*}(\mathcal {P}_{A}\cup \mathcal {P}_{B})$, which is a portion of $FCVD ^{*}(\mathcal {P}_{A})$ or $FCVD ^{*}(\mathcal {P}_{B})$. The navigation process searches all such internal edges.

During the navigation process, at each node, we pause, navigate to one child, or navigate to both children. By Lemma 23 and navigation process case 2(c), we navigate to two children if and only if *Q* straddles $(p_{1},p_{2})$. So we can charge such a branching to the straddle. The straddle is charged at most once because the cluster $Q$ can only straddle $(p_{1},p_{2})$ once by the proof of Proposition 7. Summing over all internal edges, the total number of searches is then $O(n+\textit{str}(\mathcal {P}))$.

Since the height of the tree $T_{e}$ is $O(\log n)$, the depth of search on each edge is at most $O(\log n)$. During the navigation process, we evaluate $d_c(\cdot ,Q)$ at the endpoints *u*, *v* of *e*. This can be done in $O(\log n)$ time by locating the position of the points *u*, *v* in the nearest Voronoi diagram of $Q$.

Hence, in total, the searching step for the starting points of bounded components of *M* runs in $O((n+\textit{str}( \mathcal {P}))\log ^{2} n)$ time. The construction of the binary search tree can be done in $O( ( n+\textit{str}(\mathcal {P}) ) \log (n+\textit{str}(\mathcal {P})) )=O((n+\textit{str}(\mathcal {P}))\log n)$ time as shown by Iacono et al. [19]. This completes the proof of the lemma. $\square$

Now we are ready to state the main result in this section.

### Theorem 5

Given a family of clusters $\mathcal {P}$ in the plane, the farthest color Voronoi diagram $FCVD ( \mathcal {P})$ can be constructed in $O((n+\textit{str}(\mathcal {P}))\log ^3 n)$ time, where *n* is the total number of points in $\mathcal {P}$ and $\textit{str}(\mathcal {P})$ is the total number of straddles.

### Proof

The merging step of the algorithm has two tasks. By Lemma 24, Task (1), computing starting points, takes $O((n+\textit{str}(\mathcal {P}))\log ^2 n)$ time. Task (2), tracing, can be performed in overall linear time by using the visibility-based decomposition as discussed earlier, that is, in $O(n\alpha (m)+\textit{str}(\mathcal {P}))$ time. Combining the two steps, the merge of the two sub-diagrams requires $O((n+\textit{str}(\mathcal {P}))\log ^2 n)$ time. Then solving the recurrence relation gives us the desired result. $\square$

## Concluding remarks

In this paper we presented structural properties of the farthest color Voronoi diagram (FCVD) and new, more efficient algorithms to compute it.

We showed that the complexity of the diagram is $O(n\alpha (m)+\textit{str}(\mathcal {P}))$, where *n* is the number of points, *m* is the number of colors, and $\textit{str}(\mathcal {P})$ is a parameter reflecting the number of so-called straddles between pairs of clusters. The number of straddles $\textit{str}(\mathcal {P})$ is $O(m(n-m))$ but it may also be small, even compared to *n*. Specifically, we showed that the number of unbounded faces is $O(n\alpha (m))$ and the number of bounded faces is $O(n+ \textit{str}(\mathcal {P}))$; both bounds are tight in the worst case. We also gave sufficient conditions under which the complexity of the diagram is *O*(*n*). We further showed that the complexity of the diagram can be $\Omega (n+m^2)$, even if the clusters in $\mathcal {P}$ are pairwise linearly separable. This indicates a notable difference from the closely-related Hausdorff Voronoi diagram, which is known to have *O*(*n*) complexity in this case.

On the algorithmic front, we presented algorithms to compute the diagram in $O((n+\textit{str}(\mathcal {P}))\log ^3 n)$ time, which is considerably more efficient than previous results if the number of straddles is small compared to *mn*. When the FCVD falls under the framework of abstract Voronoi diagrams, it simplifies to a tree of *O*(*n*) complexity that can be computed in optimal $O(n\log n)$ time.

One of the main contributions of this work is to show that straddles are responsible for the potential superlinear behavior of the FCVD. However, not all straddles create Voronoi vertices in the $FCVD (\mathcal {P})$; only those whose corresponding pair of disks enclose points from all colors. Straddles whose disks are empty in their interior appear as mixed vertices in the nearest color diagram of $\mathcal {P}$, and thus, these are only linearly many. Straddles whose pair of disks, each enclose points from *k* colors appear in the *order-k color Voronoi diagram* of $\mathcal {P}$. Thus, the total complexity of straddles gets distributed into different order-*k* diagrams for different values of $k, k\le m{-}1$.

Despite the progress in understanding the $$FCVD $$ and the causes for its potential quadratic behavior, we are lacking a clearly output-sensitive algorithm. Therefore, we finish by highlighting the main problem that remains open: Given a family $$\mathcal {P}$$ of clusters in the plane, can we compute the farthest color Voronoi diagram $$FCVD (\mathcal {P})$$ in output-sensitive time?

## Data Availability

No datasets were generated or analysed during the current study.

## Footnotes

1 In the preliminary version of this work [23] this bound was erroneously stated as *O*(*n*).

2 url:https://www.geogebra.org/m/rzyqx3ys.
